# Impact of *in utero* airborne lead exposure on long-run adult socio-economic outcomes: A population analysis using U.S. survey and administrative data

**DOI:** 10.1371/journal.pone.0293443

**Published:** 2023-11-22

**Authors:** H. Spencer Banzhaf, Melissa Ruby Banzhaf

**Affiliations:** 1 Department of Agricultural and Resource Economics, North Carolina State University, Raleigh, North Carolina, United States of America; 2 Property and Environment Research Center (PERC), Bozeman, Montana, United States of America; 3 National Bureau of Economic Research (NBER), Cambridge, Massachusetts, United States of America; 4 Federal Statistical Research Data Centers, United States (US) Census Bureau, Durham, North Carolina, United States of America; Sungkyunkwan University School of Social Sciences, REPUBLIC OF KOREA

## Abstract

As a neurotoxin, early exposure to lead has long been assumed to affect socioeconomic outcomes well into adulthood. However, the empirical literature documenting such effects has been limited. This study documents the long-term effects of *in utero* exposure to air lead on adult socio-economic outcomes, including real earnings, disabilities, employment, public assistance, and education, using US survey and administrative data. Specifically, we match individuals in the 2000 US Decennial Census and 2001–2014 American Community Surveys to average lead concentrations in the individual’s birth county during his/her 9 months *in utero*. We then estimate the effects of shocks to airborne lead conditional on observable characteristics, county fixed effects, county-specific time trends, and month-year fixed effects. We find a 0.5 μg/m^3^ decrease in air lead, representing the average 1975–85 change resulting from the passage of the U.S. Clean Air Act, is associated with an increase in earnings of 3.5%, or a present value, at birth, of $21,400 in lifetime earnings. Decomposing this effect, we find greater exposure to lead *in utero* is associated with an increase in disabilities in adulthood, an increase in receiving public assistance, and a decrease in employment. Looking at effects by sex, long-term effects for girls seem to fall on participation in the formal labor market, whereas for boys it appears to fall more on hours worked. This is the first study to document such long-term effects from lead using US data. We estimate the present value in 2020, from all earnings impacts from 1975 forward, to be $4.23 Trillion using a discount rate of 3%. In 2020 alone, the benefits are $252 B, or about 1.2% of GDP. Thus, our estimates imply the Clean Air Act’s lead phase out is still returning a national dividend of over 1% every year.

## Introduction

From Roman orators to modern scientists, commentators have stressed the dangers of exposure to lead, especially for developing nervous systems. Exposure to high lead levels *in utero* or in young childhood can cause low birth weight, anemia, kidney damage, and brain damage [1]. It also can affect fertility rates among women [2]. Indeed, historically lead was used as an abortifacient [3]. Even at low levels, lead exposure reduces cognitive ability as measured by IQ and school test scores and affects brain morphology [4–16]. These effects appear to be long lasting, with higher infant and preschool blood lead levels associated with lower grade-school test scores or adult brain morphology [4, 6, 8, 13, 15]. Lead exposure also causes behavioral problems such as depression, anxiety, aggressiveness, and ADHD [17–19], school suspensions and juvenile delinquency [20, 21], and criminal and other risky behavior [11, 22–24]. Given high rates of lead exposure in the US population, recent research suggests these effects may be substantial [25, 26].

Both childhood IQ and these other soft skills are, in turn, correlated with adult earnings. Accordingly, researchers have long speculated that, through these health channels, early exposure to lead could have life-long effects on socioeconomic outcomes. Over 35 years ago, the original US benefit-cost analysis of phasing out leaded gasoline estimated benefits using this logic [27]. Because there was no *direct* evidence linking lead exposure to these long-term outcomes, the analysts relied on linking two distinct bodies of evidence, a childhood lead-IQ relationship and an adult IQ-earnings relationship. From that seminal work to the present day, public health and economic analysts have continued to use this indirect methodology to estimate the costs of lead exposure [28–35]. They have used similar approaches to quantify the effects of other air pollutants as well, including other air toxics [36, 37] and fine particulates [38].

Although direct evidence of this long-term connection continues to be elusive, research has begun to fill the gap, with evidence of a relationship between lead and long-run outcomes slowly accumulating. Most of this evidence has relied on cohort-level analysis, e.g. leveraging differences in the timing of the phase out of leaded gasoline among countries or US states when looking at cohort level criminality or other unhealthy behaviors and personality traits [19, 22–24]. Individual-level studies of the long run impacts of early-life exposure to lead are still rare. In New Zealand, a cross-sectional study found blood lead at age 11 was correlated with both brain morphology and the socioeconomic score of the individuals’ adult occupations [13, 39]. In the US, children living in cities where they would have been most at risk of lead-contaminated water had lower cognitive function as older adults [40]. Of most relevance, a recent study of individuals in Sweden found that large decreases in air lead associated with the phaseout of leaded gasoline, as measured by absorption in moss, increased the probability of high school completion and decreased criminal conviction rates, especially among boys and people of lower socio-economic status [41]. Surprisingly, it found no effect on earnings, possibly because many Swedes do not complete college until their late 20s, so many were not in the labor market.

In this paper, we analyze the long-term effects of improvements in US air lead concentrations on adult socio-economic outcomes, including real earnings, disability, time working, the collection of public assistance, and education. Using confidential US Social Security records containing information on place and date of birth, we match 280,000 adults in annual 2000–2014 US Census surveys, born between 1970 and 1989, to the average air lead in their birth county in the 9 months preceding their birth date. We regress adult outcomes on these pollution measures, while controlling for individual-level characteristics (e.g., race, sex, age) as well as using panel-data methods to control for time and spatial factors. In particular, we estimate the effects of lead by leveraging the short-term departure from each county’s long-run time trend in lead levels, once national-level annual shocks are also absorbed. Thus, this study is the first to estimate such long-run effects of lead in the US using individual-level data. It is also the first to quantify economic benefits directly, rather than infer them from effects on IQ.

We find a 0.5 μg/m^3^ decrease in air lead, representing the average 1975–85 change resulting from the passage of the U.S. Clean Air Act, is associated with an increase in earnings of 3.5%, or a present value, at birth, of $21,400 in lifetime earnings. Decomposing this effect, we find greater exposure to lead *in utero* is associated with an increase in disabilities in adulthood, an increase in receiving public assistance, and a decrease in employment. Looking at effects by sex, long-term effects for girls seem to fall on participation in the formal labor market, whereas for boys it appears to fall more on hours worked. This is the first study to document such long-term effects from lead using US data. We estimate the present value in 2020, from all earnings impacts from 1975 forward, to be $4.230 Trillion using a discount rate of 3%.

## Methods

We combine restricted US Census Bureau data on adult socio-economic outcomes and birthplace information with publicly available air monitor data collected by the US Environmental Protection Agency (EPA). The resulting dataset contains individuals’ adult outcomes linked to a measure of the ambient air quality during the nine months *in utero*.

### Study population and individual linking of long-run outcomes to births

Our study population comes from restricted versions of the 2000 Decennial Census and annual American Community Surveys (ACS) from 2001–2014. Individuals in these surveys are assigned a protected-identity key (PIK), which is a unique, individual identifier that is consistent across all internal Census Bureau datasets [42]. Using this PIK, we link them to the Census Numident file, another restricted dataset. The Census Numident file contains birthdate, birth state, and birthplace (recorded as a 12-character string) for the universe of individuals with Social Security numbers, derived from Social Security Administration records.

The birthplace information comes from the application for a social security number, the SS-5 form. Historically, the applicant, or his/her parent or guardian, completes the form indicating the city, county, and state or country of birth. The Social Security administration digitizes the provided city and/or county of birth information into the 12-character birthplace string. We convert this birthplace string variable into a county FIPS code using a previously developed algorithm [43–46]. Using this procedure, we successfully matched 87% of our initial sample to a clean county and date of birth.

### Outcomes

Scientific studies have shown that lead interferes with developing nervous systems, especially the brain, leading to permanent reductions in cognitive function as well as noncognitive skills [1]. Consequently, we focus on socioeconomic outcomes that may be impacted by diminished skills, including measures of employment, disabilities, the receipt of public assistance, education, and earnings.

To capture lead’s overall effect on a variety of related socioeconomic outcomes, we aggregate the outcomes into two indices, a strategy also used in previous work [47–49]. The first index includes a set of indicators of general socioeconomic welfare, for individuals aged 30 or over. We use a minimum age of 30 in order to diminish the effects that schooling and intermittent employment may have on results measured earlier in the lifecycle. This group of outcomes includes:

- The inverse hyperbolic sine of earnings in $2014;
- the total number of different types of disabilities as collected by the ACS questionnaire;
- whether individuals reported any cognitive disability;
- whether they worked the previous year;
- whether they were unemployed (looking but unable to find work);
- whether they collected any type of public assistance income in the previous year (Social Security, Supplement Security Income, or “any public assistance or welfare payments from the state or local welfare office”);
- whether they graduated from high school; and
- whether they graduated from college.

In order to more easily and consistently interpret the effect of lead exposure, we have renormalized some outcomes so they are all consistent with the interpretation of a negative effect as detrimental (e.g., we use the *negative* of the total number of disabilities and whether an individual does *not* have any cognitive disability, is *not* unemployed, and does *not* collect public assistance). We observe these outcomes for approximately 280,000 individuals aged 30–44. (All sample sizes are rounded to the nearest thousand, in accordance with US Census disclosure rules for restricted data).

Following previous work [47–49], we then create our index by standardizing each outcome to a standard normal distribution and then average over each group of outcomes. Because all underlying variables have been reframed as desirable, the higher the index variable, the better the adult outcomes. We then use the resulting summary index as a dependent variable, representing the composite outcome.

This index is useful for testing the hypothesis that lead affects long-run socioeconomic outcomes, but to decompose it, lend it a more concrete interpretation, and facilitate quantifying dollar impacts, we also document each outcome separately. As a summary measure of overall dollar impacts, we focus especially on the inverse hyperbolic sine of real annual wage and salary earnings. This overall measure combines effects at the “extensive margin,” that is, the effect of going from zero earnings to some positive level of earnings, and effects at the “intensive margin” from greater time working or higher wages. Because earnings, conditional on being positive, are approximately log-normally distributed, using a log transformation of earnings is common practice. To accommodate zero earnings, we employ the inverse hyperbolic sine transformation instead of the log:

$$ihs\_earnings=ln\left(earnings+\sqrt{{earnings}^{2}+1}\right)$$

This measure allows us to capture the overall effect of lead on earnings through all channels. In a sensitivity analysis, we also consider ln(earnings+1).

Finally, we note that because educational attainment is highly age dependent, while at the same time different birth cohorts experienced different lead levels, in a sensitivity analysis we also consider these educational outcomes as of age 25 (which requires a different sample using only 25-year-olds). This variant isolates variation in lead while holding age constant.

The outcomes discussed above capture overall socioeconomic status, regardless of whether an individual is working. In contrast, our second index captures labor-market outcomes for individuals aged 30 or over *conditional* on working. It includes:

- Logged annual earnings (wages and salaries) in $2014;
- logged hourly wage rate in $2014;
- whether they worked the full year (at least 50 weeks);
- whether they worked full time (at least 35 hours); and
- the average number of hours worked weekly (censored at 60 hours) (hours).

These variables are derived from reported wage and salary income from the previous year and do not include self-employment earnings. We observe approximately 212,000 individuals with all of these outcomes reported.

Index 2 represents the intensity of work in the formal labor market, conditional on working, where “intensity” here is captured in both time and money. Overall earnings are of course measured in money. Differences in this outcome may reflect differences in hours worked or in the hourly compensation rate. The worked-full-year, worked-full-time, and hours-worked variables capture the amount of time worked for those that do work. Including all three variables allows the effect of lead to vary both linearly and non-linearly with usual hours worked. The hourly wage measure is calculated by dividing annual wage and salary earnings by the usual hours worked per week multiplied by the number of weeks worked in the previous year. Economists often view wage rates as a proxy for productivity. Thus, all these outcomes are various measures of how much labor-market work is performed, conditional on working.

Panel A of Table 1 gives summary statistics of all the variables that comprise Indexes 1 and 2, for the overall population and broken out by sex, using sampling weights defined in more detail below. As shown in the first row, the mean of the inverse hyperbolic sine transformation of income is 8.9, but 9.4 for men and 8.3 for women. Of course, the mean of a nonlinear transformation is not the transformation of the mean. The underlying average annual wage and salary earnings for an individual is $36,370 in 2014 dollars, including all zeroes averaged in, which would be 11.2 when transformed.

**Table 1:** Summary statistics of individual-level outcomes and regressors.

Variable	Definition	Mean (SD) Full Sample	Mean (SD) Males	Mean (SD) Females
**A. Outcomes**				
ihs_earnings	Inverse Hyperbolic Sine of annual wage and salary earnings in 2014$, including zeros. Truncated at $250,000 to maintain confidentiality, as required by US Census.	8.864 (4.526)	9.429 (4.215)	8.311 (4.745)
neg_numdis	Total number of disability types reported multiplied by negative 1	-0.1182 (0.5198)	-0.1220 (0.5223)	-0.1144 (0.5172)
nocogdis	Indicator for reporting no cognitive disabilities (serious difficulty concentrating, remembering, or making decisions)	0.9624	0.9593	0.9654
worked	Indicator for working last year even if only for a few hours	0.8358	0.8819	0.7906
not_unemp	Indicator for not unemployed (that is, employed, in the military, or out of the labor force)	0.9373	0.9342	0.9403
no_pa	Indicator for not collecting any public assistance last year (e.g., Social Security, Supplemental Security Income, public assistance or welfare).	0.9455	0.9594	0.9320
hs_grad	Indicator for high school graduate	0.9289	0.9191	0.9384
col_grad	Indicator for college graduate	0.3676	0.3271	0.4071
Index 1	Composite index of earnings_ihs, neg_numdis, nocogdis, worked, not_unemp, no_pa, HS_grad, col_grad	-0.01619 (0.5836)	0.002852 (0.5744)	-0.0348 (0.5918)
log_earnings	Ln (annual wage and salary earnings in 2014$), conditional on working. Truncated at $250,000.	10.40 (0.9574)	10.55 (0.8870)	10.24 (1.003)
log_wage	Ln(Hourly wage in 2014$), conditional on working. Truncated at values less than $1 per hour and greater than $75 per hour.	2.910 (0.6184)	2.967 (0.6053)	2.849 (0.6263)
work_fy	Indicator for worked at least 50 weeks last year, conditional on working	0.8044	0.8349	0.7718
work_ft	Indicator for usual hours worked at least 35 hours per week last year, conditional on working	0.8614	0.9190	0.7999
hours	Usual hours worked last year	40.82 (9.717)	43.11 (9.047)	38.36 (9.812)
Index 2	Composite index of log_earnings, wage, work_fy, work_ft, hours	-0.004999 (0.7289)	0.1387 (0.6526)	-0.1587 (0.7734)
hs_grad25	Indicator for high school graduate at age 25	0.9204	0.9084	0.9325
coll_grad25	Indicator for college graduate at age 25	0.3216	0.2756	0.3679
**B. Lead**	Air lead in µg/m^3^	0.5710 (0.4367)	0.5713 (0.4351)	0.5706 (0.4383)
**C. Regressors**				
male	Indicator for male respondent	0.4943	-	-
age	Age in years	32.18 (2.244)	32.17 (2.247)	32.19 (2.241)
am_ind	Indicator for American Indian or Native Alaskan race	0.008129	0.008368	0.007896
asian	Indicator for Asian race	0.02101	0.02159	0.02044
black	Indicator African-American race	0.1236	0.1142	0.1328
hawaiian	Indicator for Hawaiian or Pacific Islander race	0.0005183	0.0004841	0.0005518
white	Indicator for White race	0.7804	0.7876	0.7734
other_race	Indicator for “Other” race	0.04131	0.04265	0.04000
multirace	Indicator for multiple races	0.0250	0.02516	0.02484
hisp	Indicator for Hispanic ethnicity	0.1748	0.1737	0.1756

Table 1 also shows that the average number of disabilities reported by the sample is only 0.12 and around 96 percent do not report any cognitive disabilities. Men, on average, have more disabilities, but the differences, though statistically significant, are not substantively large. We observe greater average differences between men and women with respect to labor force attachment, which is consistent with previous findings [50]. On average, men in our sample are much more likely to have worked at all last year (88% vs 79%). Fewer men collected public assistance in the previous year than women by about three percentage points (pps) (93% vs. 96%).

The differences between men and women with respect to these outcomes are reflected in the average of Index 1. Recall that higher values of the index variable reflect more positive outcomes. The sample averages of 0.003 for men compared with -0.035 for women reflect the disparities observed in labor force attachment for men and women.

Turning to the second grouping of variables, conditional on working, we see that men tend to earn more than women both in terms of annual earnings (conditional on being positive) and in terms of the hourly wage rate (with logged wages of 2.97 and 2.85 respectively, which correspond to $19.50 and $17.29). In terms of time worked, a larger percentage of individuals work full-time (86 percent) than work the full year (80 percent). The average number of hours worked in a usual week is about 41. Men have higher values for all three variables, especially for working full time (92% vs. 80%). As with the previous index, men have higher values of Index 2 than women, on average.

### Lead exposure

Air lead measures come from the U.S. EPA’s ambient monitoring network for 1970–89. These data are available daily at precisely geocoded locations nationally. We assign these data to individuals using a three-step process. First, for each day, we find the three nearest active monitors to each Census “place” (a local geographic unit), weighting by the inverse of the distance. A place-day is valid if the nearest monitor is within 40 km. Second, we aggregate these place-day averages up to county-day averages, weighting by population. To be valid, a county-day must be composed of valid place days that represent at least 50% of the county’s population. (We also considered a coverage of 75% but found little sensitivity of our results to this alternative). These two steps construct a county-day average representative of the county’s population. Such county-level averages are common when working with long-run linkages [44]. Moreover, as discussed in the S1 Appendix, when weighted by population in this way, such county-level pollution measures are arguably more relevant than more local measures and have better statistical properties.

Finally, for each day, we constructed a fixed 9-month lagged average representing *in utero* exposure, using the following steps. First, we constructed three 89-day trimester averages, appropriately lagged from the birthdate. To be valid, each trimester average must have 12 or more valid days. This corresponds to the US EPA’s one-in-six day sampling plan combined with its criterion that 75% of days be observed. We then took the average of each trimester. If any trimester is missing based on the above criterion, we eliminate that county-birthdate. This entire process corresponds to EPA’s procedure for computing an annual average as the average of four quarters, but here for three quarters, and assures representative coverage throughout the gestational period.

**Fig 1:**
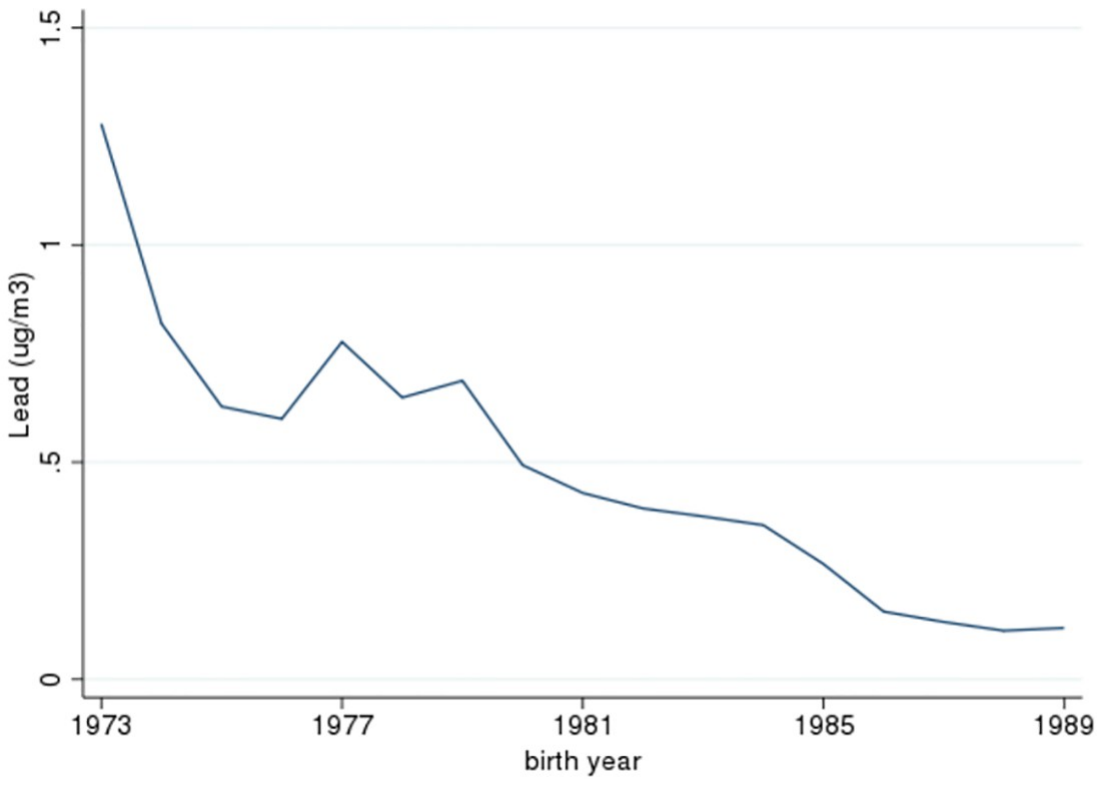
Time trends in U.S. air concentrations of lead, 1973–1989. The graph shows average weighted individual-level in utero air lead levels by year in our sample.

Summary statistics for lead exposure, for individuals in our sample, are given in Panel B of Table 1. Additionally, S1 Fig displays the density of the distribution of exposure. The table shows that the average air lead value experienced *in utero* by individuals in the sample is 0.57 µg/m^3^. There also is substantial variation in exposure, with a standard deviation of 0.44. Some of this variation is driven by the time series, as overall exposure to lead improved markedly over our study period in the United States. Fig 1 documents these improvements. The graph plots average population-weighted lead values for the entire country (whether in our sample or not), by birth year. It documents rapid improvements from 1973–5, followed by more gradual improvements. These improvements in airborne lead concentrations are largely the result of the phase-out of leaded gasoline [16]. Previous studies have found that these improvements corresponded with improvements in biomarkers of exposure. The average blood lead level (BLL) in 1- to 5-year-old US children fell from 15.0 μg/dL in 1976–1980 to 3.6 μg/dL 1988–1991, largely because of the phase-out in leaded gasoline as well as leaded paint [51, 52]. Finally, note that, as seen in S1 Fig, the distribution of exposure is highly skewed, with a long right-hand tail.

### Covariates

The ACS and Decennial surveys also provide demographic control variables at both the individual and the county level. Exogenous individual-level variables include sex, race dummies, Hispanic ethnicity, and age, all as self-reported at the time the outcome is observed. (Note that we omit survey-year because it is not linearly independent of year-month of birth and age). The model contains interactions of these demographic controls as well as main effects. Panel C of Table 1 provides summary statistics of these covariates. Most of these controls are not significantly different between the male and female subsamples.

Ideally, we would include additional information about the individual’s parents, such as education or income, as regressors. Unfortunately, we do not observe that information. However, our main specifications do include county-level average demographics at the year of birth to control for any differences in “neighborhood” effects that may affect long-term outcomes, and which are correlated with parents’ values for these variables. We construct these county-level control variables from the 1970, 1980, and 1990 Decennial Censuses. We calculate county level averages for each Decennial year and then linearly interpolate the measures between Decennial years to produce annual averages. These county-level controls include:

- Percent of households with any children
- Percent of households with a female head of household
- Average household size
- Percent of households that are rural
- Percent of white individuals
- Percent of black individuals
- Percent of Hispanic individuals
- Percent of married individuals
- Percent of never married individuals
- Percent employed
- Percent of high school graduates
- Percent of college graduates
- Percent of individuals on public assistance
- Per capital real personal income
- Employment per capita.

In sensitivity analyses, we also consider models that omit these variables.

### Statistical modelling

We use the following panel model to identify the effects of lead on our long-run socio-economic outcomes:

$$Y_{i,c,d}={\beta *Lead}_{c,d}+x_{i}^{'}\gamma +{\bar{z}}_{c,y}^{'}\delta +\theta_{c}+\mu_{c}*{date}_{d}+\varphi_{m,y}+\varepsilon_{i,c,d},$$

where *Y*_*i*,*c*,*d*_ is one of the outcome measures for person *i* born in county *c* on day *d; β* is the parameter of interest for the effect of the 9-month average of lead lagged from day *d* in county *c*; ***x*** is a vector of exogenous individual characteristics; $\bar{z}$ is a vector of county-level average characteristics for county *c* in birth year *y*; *θ* is a set of county-of-birth fixed effects, which capture time-invariant geographic factors including long-run average observed and unobserved population characteristics; μ_c_*date_*d*_ represents county-specific linear time trends, which capture any trends in the county correlated with trends in lead; *φ* is a set of month (*m*) by-year (*y*) birth effects, which capture effects of national cohorts. The error *ε*_*i*,*c*,*d*_ is clustered by state.

This estimation strategy relies on identifying the effects on long-term socio-economic outcomes using local deviations in pollution from long-term county trends as well as from national shocks common to those born in the same month and year. Any unobserved socioeconomic factors that are correlated with county differences and county-level time trends are thus captured by these fixed effects. This design controls for households moving (or “sorting”) into low- and high- pollution areas, as the fixed effects absorb locational decisions. That is, households that differ in unobserved ways may choose to live in different areas with systematically different lead levels and long-term trends, so long as they do not do so based on the lead levels they will experience during a pregnancy. This design is plausible because it is unlikely that, when moving, individuals could predict how lead levels at the time of a future pregnancy could differ from the county’s trendline. The design also controls for other public investments that may have coincided with phaseouts in lead. For example, if the phaseouts coincided with educational investments, those investments would have affected other school-age children long before they affected those just born at the time, and hence would be captured in our fixed effects and time trends.

Finally, our main models weight the observations to account for Census sampling probabilities and the probability of being successfully matched to a county of birth. As noted above, we were able to match 87% of the initial sample. Individuals may be unmatched because the PIK is missing, because two respondents in the same survey year receive the same PIK, or because a PIKed individual cannot be matched to a unique county of birth due to data quality issues in the 12-character string variable. Unfortunately, when county of birth is missing, it is potentially missing in non-random ways [53]. To control for the selection into our sample, we estimate the probability of having a unique county of birth on the original sample using a probit model, calculate inverse probability weights for each individual, and use these weights, multiplied by Census’s sample weights, to control for the non-random selection of our analysis sample. We also control for observable characteristics of individuals and counties. In addition to these main models, we also consider non-linear functions of lead and models with different effects by sex, as well as additional sensitivity analyses described in more detail below.

## Results

### Main results for linear models

**Fig 2:**
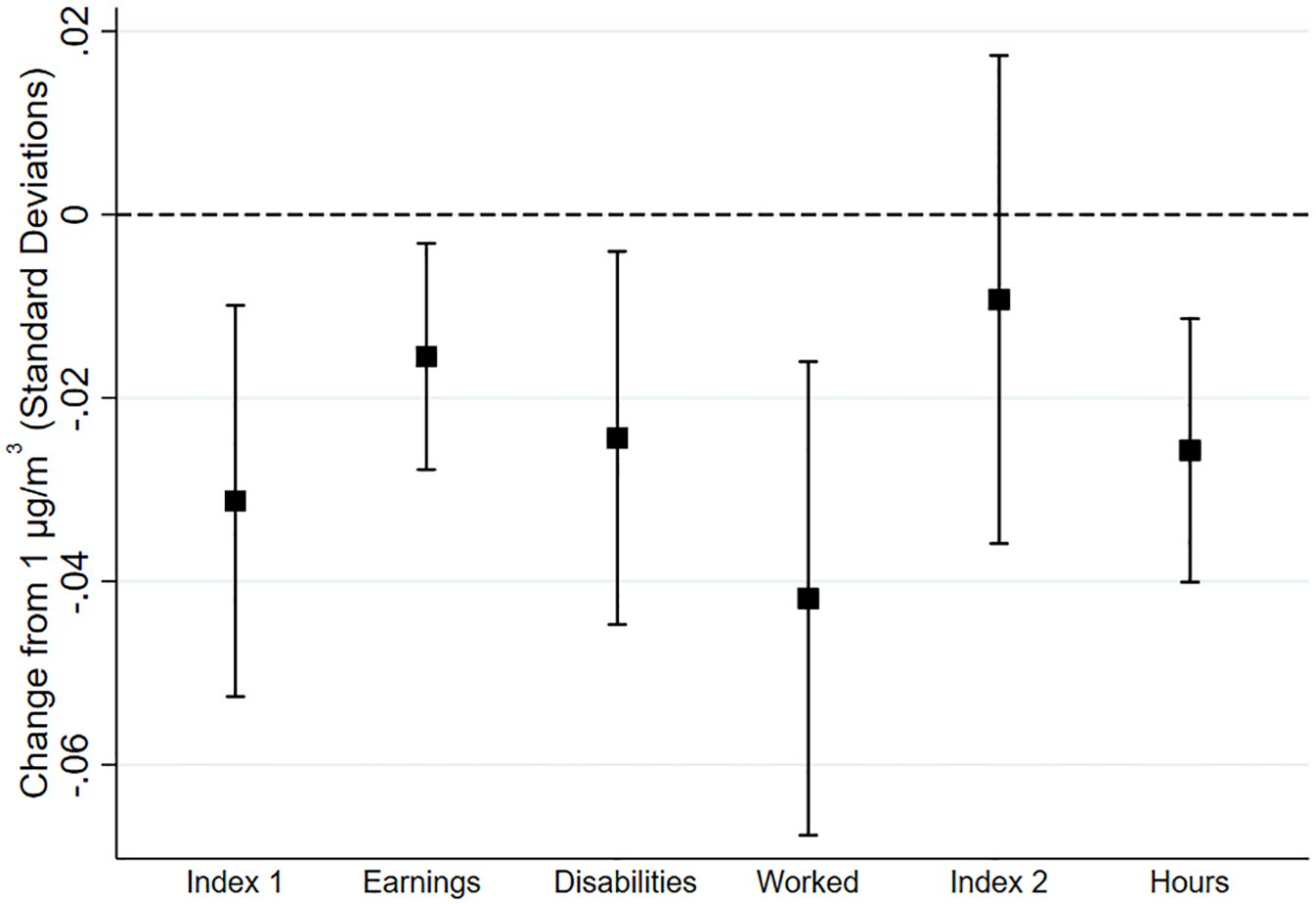
Selected point estimates and confidence intervals. The graph shows the effects of exposure to an additional 1 μg/m^3^ of atmospheric lead, while *in utero*, on each outcome index, as well as selected index components.

Fig 2 summarizes our results. It displays the effects of exposure to an additional 1 μg/m^3^ of atmospheric lead, while *in utero*, on each index, as well as selected components. To facilitate comparisons across outcomes, all effects in the graph are measured in standard deviations. The figure shows that lead has significantly negative effects on Index 1 and some of its components, including earnings, disabilities, and whether the person worked last year. It does not have a significant effect on Index 2, though it does on the component hours worked.

Table 2A and 2B presents additional details, including coefficients and standard errors for the indices and all components. Each column of the tables represents an outcome (and a separate regression). The rows show, respectively, the estimated coefficient, the standard error clustered by state, p-values, and the R^2^ from the model.

**Table 2:** **A.** Outcomes for General Index, Linear Model. **B.** Outcomes Conditional on work, Linear Model.

**A**									
Dependent vars:	**Index 1 (Std Dev.)**	Inverse Hyperbolic Sine of Earnings	Negative of the No. of Disabilities	No Cognitive Disability	Worked Last Year	Not Unemployed	No Public Assistance	Graduate from HS	Graduated from Col.
Lead	-0.03124**	-0.07010**	-0.01267*	-0.005565**	-0.008516**	-0.004156*	-0.004706*	-0.003134	-0.0004416
SE	(0.01297)	(0.03396)	(0.006428)	(0.002686)	(0.003193)	(0.002441)	(0.002556)	(0.003911)	(0.005704)
p-val	0.0198	0.044	0.054	0.044	0.010	0.095	0.072	0.427	0.939
Adj p-val	n/a	0.105	0.105	0.105	0.087	0.122	0.113	0.146	0.307
R^2^	0.0505	0.0218	0.0132	0.0117	0.0403	0.0214	0.0252	0.0414	0.0902
N (rounded)	280000	280000	280000	280000	280000	280000	280000	280000	280000

**B**						
Dependent vars:	**Index 2 (Std Dev.)**	Ln Earnings, if >0	Ln Hourly wage, if >0	Worked full year (if working)	Worked full time (if working)	No. Weekly Hrs Usually Worked (if working)
Lead	-0.00927	-0.0006396	0.01034	-0.007473	-0.001974	-0.2499***
SE	(0.01618)	(0.01496)	(0.01135)	(0.005980)	(0.004229)	(0.08486)
p-val	0.569	0.966	0.367	0.217	0.643	0.005
Adj p-val	n/a	1.000	0.957	0.770	1.000	0.026
R^2^	0.0737	0.0697	0.0731	0.0243	0.0459	0.0774
N (rounded)	212000	212000	212000	212000	212000	212000

Each grid cell represents a separate regression. Regressions include county-specific time trends, county-year demographic averages, month-of-birth fixed effects, race, sex, and age. Weights account for Census sampling probabilities and the probability of being matched to unique birth county

The coefficient in the first column of Table 2A reveals that lead exposure has a statistically significant effect on our general index. Specifically, a 1 μg/m^3^ increase in exposure to airborne lead, while *in utero*, is associated with a decrease in the index of about 0.03 standard deviations. The result is statistically significant (*p*-value = 0.02).

To explore which components are driving the effects on the overall index and to give a more socio-economically meaningful interpretation to this summary-level result, the remaining columns of Table 2A show the effects on the specific outcomes underlying the index. In this exercise, we are not so much testing a series of separate hypotheses about discrete outcomes (which would increase the chance of at least one Type-I error), as exploring *which* subcomponents are driving the results for our pre-determined index. Consequently, conventional standard errors and p-values are appropriate in this case. However, as an alternative, we also report p-values adjusted for multiple hypothesis tests [54, 55].

The estimated coefficient on the inverse hyperbolic sine of earnings, an outcome that encapsulates in one summary measure labor-market outcomes at both the extensive margin (working or not working) and intensive margin (hours worked and hourly wages), implies that a 1 μg/m^3^ increase in lead is associated with a 7.0%, or 0.02 SD, decrease in the measure (*p*-value = 0.04). To put this in context, it implies a 0.5 μg/m^3^ decrease in air lead, representing the average change from 1975 to 1985, is associated with an average increase in annual earnings of 3.5%. In a sensitivity analysis, we also considered a simple transformation of ln(earnings + 1), finding a similar effect of 6.6% (*p*-value = 0.04).

Also noteworthy is the finding that a 1 μg/m^3^ increase in lead is associated with an average increase of 0.013 disabilities or, equivalently, one disability per 79 people (*p*-value = 0.05). It also is associated with a 0.9 pp increase in the probability of not working at all in the previous year (*p*-value = 0.01) and 0.5 pp increase in the probability of receiving public assistance (*p*-value = 0.07). With a total US population of about 87m people ages 20–44 today, the 0.5 μg/m^3^ 1975–85 decrease in airborne lead translates into about 551,000 disabilities, 370,000 jobs and 205,000 cases of public assistance.

On the other hand, we find only small and statistically insignificant effects of exposure to airborne lead on the probability of graduating from high school or college. In a sensitivity analysis, we also estimated these effects only in a sample of people exactly age 25, again finding no meaningful effects (see S1 Table).

Table 2B shows the detailed results for the index of outcomes conditional on working. As seen in the first column, a 1 μg/m^3^ increase in airborne lead is associated with a statistically insignificant decrease in the index of about 0.01 standard deviations. As discussed above, we found economically meaningful effects on disabilities and the probability of working at all, so it appears that those effects at the “extensive margin” are a more important driver of the overall results than these effects at the “intensive margin” (productivity conditional on working). Similarly, most individual components in Table 2B are negative but not significant, with the exception of hours worked, where we find that a 1 μg/m^3^ increase in airborne lead is associated with about a quarter of an hour less time working per week (*p*-value < 0.01). In a population of 87m, working 50 weeks per year, this translates to 544 million labor-hours gained annually from the 0.5 μg/m^3^ improvement from 1975–85, equivalent to an additional 272,000 full-time jobs.

For both indices, we conducted two additional sensitivity analyses. First, we estimated unweighted models, as a sensitivity analysis of our weighting procedure. Most of the results using raw data are very similar in magnitude to those from the weighted model (see S2 and S3 Tables). Additionally, we omitted the county-level demographic controls, relying only on individual controls and fixed effects. Again, the point estimates and standard errors are very similar using this approach (see S4 and S5 Tables).

### Nonlinear models

Our linear models identify the marginal effects of airborne lead averaged over the distribution of lead concentrations. As the probability density function (pdf) graphed in S1 Fig shows, the distribution is highly variable across time and space as well as skewed. *In utero* exposure to airborne lead ranges from approximately 0.08 to 0.30 μg/m^3^ at the 1^st^ to 25^th^ percentiles and 0.72 to 2.1 μg/m^3^ at the 75^th^ to 99^th^. That wide range raises the question of potential nonlinear effects, with marginal effects possibly either attenuating at higher lead levels or increasing as thresholds are crossed. The literature generally has found that the marginal effects of blood lead levels on test scores are greater at lower levels [5, 10], but recent work in Sweden finds larger marginal effects of air lead at higher levels [40], though the overall distribution is much lower there than in the US.

To further explore these issues, we estimated models with quadratic and cubic functions of lead as regressors, while still including the same fixed effects and other controls. S6–S9 Tables show the estimated regression coefficients for these models, as well as tests of joint significance. Based on these models, Table 3A and 3B show the marginal effects at three points in the lead distribution, the 5^th^ percentile (0.18 μg/m^3^), median (0.45 μg/m^3^), and 95^th^ percentile (1.29 μg/m^3^). It displays results in two groups of rows, corresponding to a quadratic and cubic model, respectively.

**Table 3:** **A.** Marginal Values, for Outcomes in General Index, at Three Points in the Distribution, Polynomial Models. **B.** Marginal Values, for Outcomes Conditional on Working, at Three Points in the Distribution, Polynomial Models.

**A**									
Dependent vars:	**Index 1 (Std Dev.)**	Inverse Hyperbolic Sine of Earnings	Negative of the No. of Disabilities	No Cognitive Disability	Worked Last Year	Not Unemployed	No Public Assistance	Graduate from HS	Graduated from Col.
Quadratic Model									
5^th^ percentile	-0.03753**	-0.1576***	-0.0155*	-0.0064	-0.0120*	-0.0056	-0.0057**	-0.0014	0.0060
SE	(0.0182)	(0.0584)	(0.0086)	(0.0039)	(0.0061)	(0.0042)	(0.0028)	(0.0046)	(0.0086)
p-val	0.045	0.010	0.078	0.107	0.055	0.189	0.047	0.762	0.489
Adj p-val	n/a	0.083	0.158	0.172	0.147	0.184	0.147	0.336	0.265
Median	-0.03667**	-0.1456**	-0.0151*	-0.0063*	-0.0115**	-0.0054	-0.0056**	-0.0016	0.0051
SE	(0.0171)	(0.0546)	(0.0081)	(0.0037)	(0.0057)	(0.0038)	(0.0027)	(0.0044)	(0.0081)
p-val	0.037	0.010	0.068	0.095	0.049	0.162	0.043	0.718	0.532
Adj p-val	n/a	0.091	0.136	0.151	0.13	0.156	0.13	0.275	0.275
95^th^ percentile	-0.0341**	-0.1087**	-0.0139**	-0.0060*	-0.0100**	-0.0048	-0.0052**	-0.0024	0.0024
SE	(0.0144)	(0.0430)	(0.0067)	(0.0031)	(0.0044)	(0.0030)	(0.0025)	(0.0040)	(0.0067)
p-val	0.022	0.015	0.043	0.059	0.027	0.116	0.043	0.551	0.722
Adj p-val	n/a	0.095	0.095	0.095	0.095	0.095	0.095	0.187	0.221
Cubic Model									
5^th^ percentile	-0.01936	-0.1398*	0.0012	-0.0051	-0.0055	-0.0143*	-0.0051	0.0083	0.0155
SE	(0.0255)	(0.0801)	(0.0156)	(0.0048)	(0.0086)	(0.0076)	(0.0051)	(0.0081)	(0.0117)
p-val	0.451	0.087	0.939	0.293	0.525	0.066	0.322	0.311	0.191
Adj p-val	n/a	0.536	0.754	0.536	0.754	0.536	0.536	0.536	0.536
Median	-0.02416	-0.1333*	-0.0036	-0.0054	-0.0070	-0.0114*	-0.0051	0.0051	0.0117
SE	(0.0202)	(0.0683)	(0.0122)	(0.0040)	(0.0072)	(0.0062)	(0.0038)	(0.0058)	(0.0099)
p-val	0.237	0.057	0.769	0.183	0.336	0.072	0.186	0.384	0.243
Adj p-val	n/a	0.405	0.625	0.405	0.49	0.405	0.405	0.49	0.412
95^th^ percentile	-0.03615***	-0.1107***	-0.0158**	-0.0061*	-0.0108***	-0.0038	-0.0052*	-0.0035	0.0013
SE	(0.0135)	(0.0399)	(0.0066)	(0.0032)	(0.0039)	(0.0032)	(0.0027)	(0.0040)	(0.0056)
p-val	0.010	0.008	0.021	0.062	0.008	0.241	0.060	0.386	0.817
Adj p-val	n/a	0.033	0.043	0.067	0.033	0.137	0.067	0.199	0.443

**B**						
Dependent vars:	**Index 2 (Std Dev.)**	Ln Earnings, if >0	Ln Hourly wage, if >0	Worked full year (if working)	Worked full time (if working)	No. Weekly Hrs Usually Worked (if working)
Quadratic Model						
5^th^ percentile	-0.02017	-0.0137	-0.0052	-0.0154***	0.0011	-0.1549
SE	(0.0166)	(0.0159)	(0.0114)	(0.0054)	(0.0068)	(0.1402)
p-val	0.230	0.393	0.650	0.006	0.872	0.275
Adj p-val	n/a	1.000	1.000	0.033	1.000	1.000
Median	-0.01866	-0.0119	-0.0031	-0.0143***	0.0007	-0.1680
SE	(0.0159)	(0.0152)	(0.0107)	(0.0051)	(0.0065)	(0.1333)
p-val	0.246	0.437	0.773	0.007	0.915	0.214
Adj p-val	n/a	1.000	1.000	0.038	1.000	0.746
95^th^ percentile	-0.01399	-0.0063	0.0036	-0.0109**	-0.0006	-0.2085*
SE	(0.0143)	(0.0131)	(0.0089)	(0.0044)	(0.0056)	(0.1147)
p-val	0.333	0.633	0.688	0.017	0.915	0.075
Adj p-val	n/a	1.000	1.000	0.092	1.000	0.178
Cubic Model						
5^th^ percentile	-0.0332	-0.0280	-0.0036	-0.0211*	-0.0091	-0.0755
SE	(0.0269)	(0.0259)	(0.0182)	(0.0116)	(0.0114)	(0.2253)
p-val	0.223	0.285	0.844	0.075	0.429	0.739
Adj p-val	n/a	1.000	1.000	0.601	1.000	1.000
Median	-0.02771	-0.0217	-0.0020	-0.0182**	-0.0064	-0.1134
SE	(0.0210)	(0.0207)	(0.0152)	(0.0089)	(0.0089)	(0.1870)
p-val	0.193	0.300	0.896	0.046	0.475	0.547
Adj p-val	n/a	1.000	1.000	0.301	1.000	1.000
95^th^ percentile	-0.01235	-0.0045	0.0033	-0.0102**	0.0006	-0.2184*
SE	(0.0148)	(0.0134)	(0.0084)	(0.0048)	(0.0054)	(0.1133)
p-val	0.408	0.738	0.696	0.039	0.912	0.060
Adj p-val	n/a	1.000	1.000	0.176	1.000	0.176

Each column and each panel represents a separate regression. For each regression, the marginal effects of lead are shown at the 5^th^, 50^th^, and 95^th^ percentiles of the lead distribution. Regressions include county-specific time trends, county-year demographic averages, month-of-birth fixed effects, race, sex, and age. Weights account for Census sampling probabilities and the probability of being matched to unique birth county.

As seen in the first column of Table 3A, the overall index tends to show effects similar to the simple linear model. The quadratic model is still very linear, with statistically significant effects of lead at all points in the distribution. In the cubic model, the point estimates for the marginal effects are highest at the highest lead concentrations, but they are not statistically different from those at lower concentrations.

**Fig 3:**
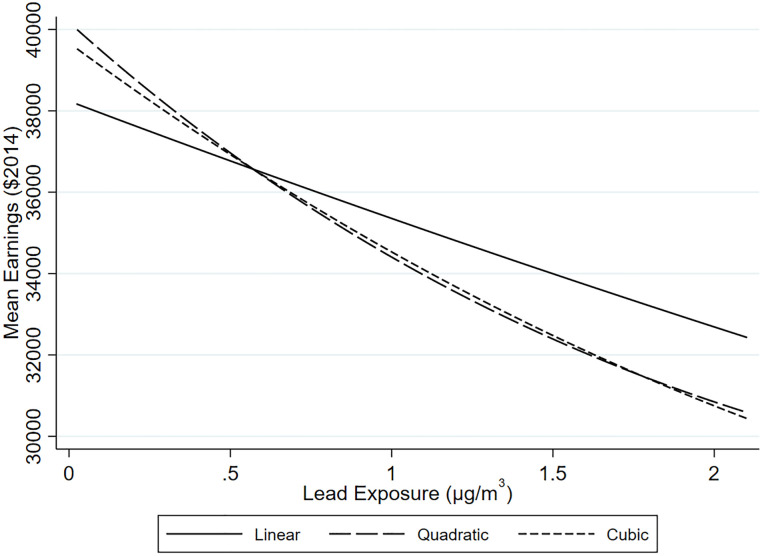
Nonlinear effects on earnings. This graph depicts effect of lead on earnings when the effect is modeled as a linear, quadratic, and cubic relationship.

Column two shows the effects of lead on the inverse hyperbolic sine of earnings is negative and statistically significant at all three points in the distribution. This component appears to exhibit the most nonlinearity. In the case of this outcome, we have some sign of attenuation, with the greatest marginal effects at lower concentrations. Fig 3 displays the quadratic and cubic relationships at various levels of lead exposure, comparing them to the linear model of the previous subsection.

In Table 3B, the first column shows the effects for Index 2 in the quadratic and cubic models. It appears fairly linear and continues to be insignificant at all points in the distribution, as with the linear model. Most of the components are also insignificant. However, there are now statistically significant effects for the working-full-year component.

### Results by sex

**Fig 4:**
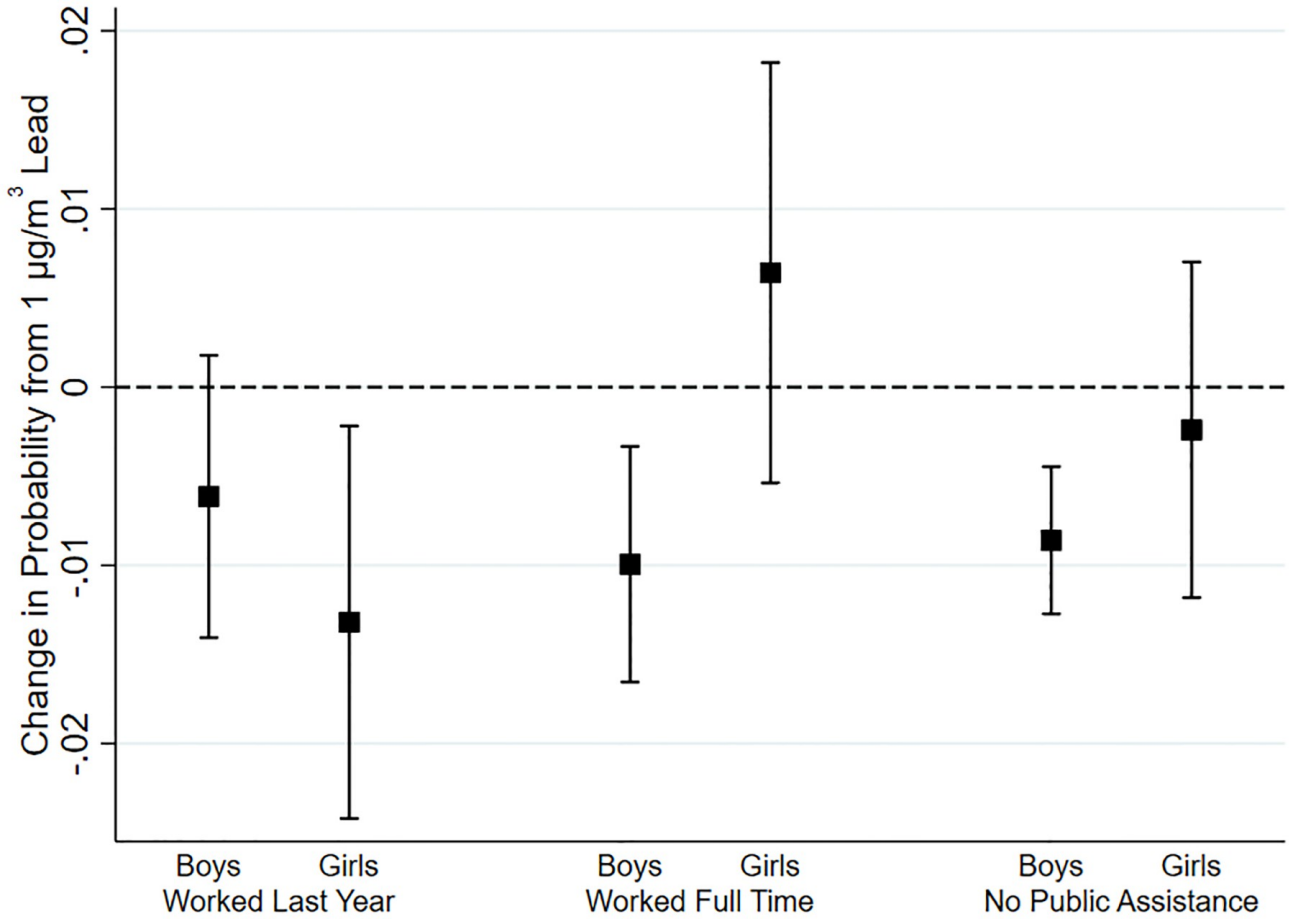
Effects by sex. This graph displays the effects of a change in lead exposure of 1 μg/m^3^ on the change in probability of select outcomes for boys and girls.

Several studies have found differing effects of lead exposure by sex, with boys appearing to be especially vulnerable [6, 20, 23, 40]. To test for potential sex differences in the long-term effects of lead on socio-economic outcomes, we estimated separate models for girls and boys. Fig 4 summarizes some of our most salient findings. Table 4A and 4B display the results in detail. Looking at the general index, we find similar point estimates for girls and boys, at about 0.035 standard deviations of the index from a 1 μg/m^3^ increase in lead exposure (*p*-value = 0.09 and 0.03 respectively).

**Table 4:** **A.** Outcomes for General Index, Linear Model, by Sex. **B.** Outcomes Conditional on Working, Linear Model, by Sex.

**A**									
Dependent vars:	**Index 1 (Std Dev.)**	Inverse Hyperbolic Sine of Earnings	Negative of the No. of Disabilities	No Cognitive Disability	Worked Last Year	Not Unemployed	No Public Assistance	Graduate from HS	Graduated from Col.
Girls									
Lead	-0.0346*	-0.1410***	-0.01938*	-0.008504**	-0.01319*	0.002187	-0.002396	-0.001307	-0.002624
SE	(0.01996)	(0.04784)	(0.01129)	(0.004023)	(0.006698)	(0.002840)	(0.005727)	(0.003660)	(0.007908)
p-val	0.089	0.005	0.092	0.040	0.055	0.445	0.678	0.723	0.741
Adj p-val	n/a	0.041	0.171	0.146	0.146	0.553	0.590	0.590	0.590
R^2^	0.0526	0.0311	0.0210	0.0185	0.0285	0.0293	0.0347	0.0487	0.0974
N (rounded)	142000	142000	142000	142000	142000	142000	142000	142000	142000
Boys									
Lead	-0.03566**	-0.02726	-0.006198	-0.003267	-0.006136	-0.01044***	-0.008596***	-0.006327	-0.001963
SE	(0.01571)	(0.05973)	(0.007873)	(0.002831)	(0.004817)	(0.003615)	(0.002513)	(0.008563)	(0.009060)
p-val	0.028	0.650	0.435	0.254	0.209	0.006	0.001	0.464	0.829
Adj p-val	n/a	1.000	0.864	0.616	0.616	0.021	0.011	0.864	1.000
R^2^	0.0649	0.0544	0.0211	0.0195	0.0588	0.0282	0.0232	0.0495	0.0838
N (rounded)	138000	138000	138000	138000	138000	138000	138000	138000	138000

**B**						
Dependent vars:	**Index 2 (Std Dev.)**	Ln Earnings, if >0	Ln Hourly wage, if >0	Worked full year (if working)	Worked full time (if working)	No. Weekly Hrs Usually Worked (if working)
Girls						
Lead	0.0283	0.02781	0.02738	0.01113	0.00642	-0.1545
SE	(0.02950)	(0.03000)	(0.01948)	(0.01172)	(0.00717)	(0.11230)
p-val	0.342	0.358	0.166	0.347	0.375	0.175
Adj p-val	n/a	0.600	0.600	0.600	0.600	0.600
R^2^	0.0401	0.0508	0.0749	0.0294	0.0310	0.0274
N (rounded)	104,000	104,000	104,000	104,000	104,000	104,000
Boys						
Lead	-0.04165***	-0.02584**	-0.003528	-0.02422***	-0.009938**	-0.2996**
SE	(0.01346)	(0.01274)	(0.00843)	(0.00591)	(0.00402)	(0.13920)
p-val	0.003	0.048	0.677	0.000	0.017	0.036
Adj p-val	n/a	0.045	0.157	0.001	0.036	0.045
R^2^	0.0578	0.0675	0.0761	0.0277	0.0293	0.0383
N (rounded)	108,000	108,000	108,000	108,000	108,000	108,000

Each grid cell represents a separate regression. Regressions include county-specific time trends, county-year demographic averages, month-of-birth fixed effects, race, sex, and age. Weights account for Census sampling probabilities and the probability of being matched to unique birth county.

However, this similarity for the overall index masks substantial sex differences in the composition of the effect. Notably, lead significantly affects the future earnings of girls, with every μg/m^3^ increase in lead exposure decreasing later adult earnings by 14% (*p*-value < 0.01). Conceptually, these effects on earnings can in turn be explained by other components of the two indices, such as whether one works at all, how much, and at what wages. Looking across other outcomes, it appears most of this effect for girls is driven by future labor-market participation, with each additional 1 μg/m^3^ increase in lead exposure reducing the probability of working in the past year by 1.3 pp. (*p-*value = 0.06). It similarly affects the number of disabilities (1.9 per μg/m^3^, *p-*value = 0.09) and presence of a cognitive disability (0.9 p.p. per μg/m^3^, *p-*value = 0.04). In contrast, conditional on working, there is little evidence that, for girls, lead has an effect on working full time or hours worked.

Boys show almost the opposite pattern. They experience a weaker relationship with future earnings (2.7%, *p*-value = 0.65) and disabilities, but stronger relationships between unemployment (1.0 p.p. per μg/m^3^, *p*-value < 0.01) and public assistance (0.9 p.p. per μg/m^3^, *p*-value < 0.01). Moreover, unlike with girls, lead is associated with much larger effects at the intensive margin, that is, how much they work conditional on working. Indeed, the estimated coefficient on Index 2 is about 1.5 times that for girls and now highly significant statistically as well as economically (0.042 SDs per μg/m^3^ vs 0.028 for girls, *p*-value < 0.01). Within this index, we find large effects on a wide range of indicators, including logged earnings conditional on working (2.6% per μg/m^3^, *p*-value = 0.05), working the full year (2.4 p.p. per μg/m^3^, *p*-value < 0.01), working full time (1.0 p.p. per μg/m^3^, *p*-value = 0.02), and hours worked (0.30 per μg/m^3^, *p*-value = 0.04).

### The economic benefits of reduction in lead exposure

As noted above, previous estimates of the benefits of reductions in lead exposure have relied on an indirect methodology, first using a relationship between lead and IQ points, then IQ points and earnings [27–35]. Our research provides a unique opportunity to estimate such economic benefits directly, integrating our new empirical estimates on the long-term relationships between lead exposure and earnings into benefit calculations.

To fix ideas, we consider the impacts of a permanent improvement in air lead concentrations of 0.5 μg/m^3^, phased in linearly between 1975 and 1985. (Thus, in 1976, we assume there was a uniform improvement of 0.05 μg/m^3^, in 1977 an improvement of 0.1 μg/m^3^, etc). This simulation captures, at least heuristically, the 1975–85 improvement seen in our data, resulting from the Clean Air Act, which phased out leaded gasoline in two primary steps at 1979 and 1985.

We compute the cumulative benefits up to 2020 in four steps. First, beginning in 1992, when individuals born in 1976 first enter the labor market, and continuing to 2020, we obtained, for each year, the total number of people alive and of each age, for ages 16 to 45, from various US Census data sets depending on the year (see S1 Appendix for details). Note age 16 is the youngest for which employment and wage data are available, and age 44 is the oldest age in 2020 for someone born in 1976. Second, we obtained total wages and salaries (“earnings”) for the United States in each year from the US National Income and Product Accounts (NIPA), which is the most reliable source of the totals. Third, using estimated average earnings by age in each year from the US Census, together with the total number of people of each age in each year from step one, we allocated the total earnings from each year’s NIPA to people of each age. Thus, relative earnings across ages come from the US Census, but the totals are calibrated to the NIPA data. (See S1 Appendix for details).

Lastly, we combine the assumed change in lead for each birth cohort (0.05 for the 1976 cohort to 0.5 μg/m^3^ for 1985 and later cohorts), the estimated 7.01% change in earnings per μg/m^3^ (Table 2A), and the total earnings of people age *a* in year *t*, *E*_*a*,*t*_. By summing across people and years and discounting *forward* from the year of the effect to 2020 at discount rate *i*, we obtain total benefits. That is, if we denote the simulated change in lead for each birth cohort as Δ*L*_*a*,*t*_ (where the cohort is age *a* in year *t*), then we calculate total benefits *B* = ∑_*t*_∑_*a*_ 0.0701 * Δ*L*_*a*,*t*_ * *E*_*a*,*t*_ * (1 + *i*)^2020-*t*^. We use discount rates of *i* = 0%, 3%, and 6%, as a reasonable range.

As summarized in Table 5, we estimate the present value in 2020, from all earnings impacts from 1975 forward, to be $4.230 Trillion using a discount rate of 3%. This figure falls to $3.189 T undiscounted and rises to $5.823 T when discounted at 6%. In 2020 alone, the benefits are $252 Billion, or about 1.2% of GDP. Thus, our estimates imply the Clean Air Act’s lead phase out is still returning a national dividend of over 1% every year.

**Table 5:** Economic benefit calculations.

Economic Benefits	*i* = 0%	*i* = 3%	*i* = 6%
Total benefits 1981–2020, for people born 1975–- 2004.	$3.189 Trillion	$4.230 Trillion	$5.823 Trillion
Benefits just in 2020, for people born 1975–- 2004. (As pct of GDP)	$252 Billion (1.20%)	$252 Billion (1.20%)	$252 Billion (1.20%)
Present Value at birth, for individual born in 1985		$21,400	
—Using US EPA (1997) procedure [30]		$5,900	
—Using Shea et al.’s (2020) preferred procedure [35]		$12,300	
—Using Gould’s (2009) procedure [33]		$23,400	

All figures in 2020 dollars.

We can also put these estimates in perspective by comparing them to previous results. Using previous methods [56, 57], we forecast the present value of *lifetime* earnings using a discount rate of 3%. We estimate this value to be $609,700 per person (see S1 Appendix for details). For somebody born in 1985 or later, receiving the full 0.5 μg/m3 improvement in air lead in our simulation, our estimated impact of 7.01% translates to a present value, at birth, of $21,400 per capita. In contrast, the indirect methods used by US EPA [30], Shea et al. [35], and Gould [33] estimated lifetime per capita impacts of $5,900, $12,300, and $23,400 respectively. Thus, our empirical estimates support the high end of the range previously estimated from indirect methods.

## Discussion

This paper finds critical individual-level evidence on the long-term links among lead, cognitive development, and observed socio-economic outcomes. Specifically, it finds statistically significant effects on an index of socio-economic outcomes, including earnings, disabilities, employment status, and receipt of public assistance, but not educational outcomes. Nonlinear models generally fail to reject the simpler assumption of linearity, although there is some limited evidence of attenuation on earnings.

These average effects appear to mask important heterogeneity by sex. We find the point estimate for lead’s effect on future earnings is substantially higher for girls than for boys, driven largely by participation in the labor market. On the other hand, the estimated effects on future earnings conditional on working, working the full year or full time, and hours worked is higher for boys. These patterns are consistent with previous findings that women exhibit less labor force attachment than men [50].

These results are probably conservative. Our research design identifies causal effects only from short-term fluctuations in *in utero* exposure, relative to the overall long-run exposure throughout life, including early childhood. However, the long-run decline in air lead would affect total airborne exposure over the lifetime. It also would be expected to reduce the deposition of lead in the soil, which could have compounding effects over time through additional channels besides respiration.

Our findings largely corroborate earlier findings from an individual-level study in Sweden [40]. However, it differs from that study in finding robust evidence of effects on long-run earnings. This may be because we observe outcomes over a longer time frame than that study, because of other differences between Sweden and the United States in demographic composition and the social and economic environment, or because we use actual air-lead measures rather than proxies from moss.

More generally, this research is part of a growing literature examining early exposure to air pollution and later-life outcomes. The “fetal origins hypothesis” posits a biological mechanism through which *in utero* health can persist through adulthood [58]. Initially focused on health outcomes, it has come to encompass other dimensions of well-being, such as human capital accumulation, labor market outcomes, and welfare dependency [59–62]. Most evidence in support of this hypothesis has been indirect. For example, a large literature suggests that pollution exposure in early childhood affects children’s health [63–67] while an equally large literature suggests that childhood health in turn has persistent human capital impacts [68–73]. Recently, research has begun to find an association between exposure to particulate air pollution in early childhood and adult labor force participation, earnings, and college completion [44, 74]. It also has suggested that these human capital effects of pollution may be transmitted across generations [75]. Our research on lead exposure complements this wider literature.

However, the broadest insight of our work might be on the importance of long-term policy evaluations. In the case of the phase-out of leaded gasoline, our research shows it had substantial economic benefits that continues to pay dividends decades later through higher earnings, reductions in disability, and lower take-up of public assistance. Using estimates of lifetime earnings, our estimates imply the 0.5 μg/m^3^ decrease in US air lead is associated with a present value, at birth, of about $21,400 per capita—a substantial figure. Benefits to date total some $4.230 T from labor-market impacts alone. Again, from an economic point of view, these results are probably conservative. They include only earnings, but not the losses from the underlying health effects themselves. Thus, the justification for the Clean Air Act’s lead reductions and, presumably, other lead abatement programs for water or paint, appears stronger than ever.

Future research should focus on a better understanding of the socio-economic mechanisms by which lead impacts earnings in the U.S., and why the results differ between men and women. It also might focus on how these mechanisms differ internationally in different institutional settings.

## Supporting information

S1 Fig Probability density function of individual lead exposures. The figure shows a kernel density of individual-level air lead exposures throughout the sample. To satisfy the Census Bureau’s disclosure requirements, the lower and upper 5% tails are omitted. The Census Bureau’s Disclosure Review Board and Disclosure Avoidance Officers have reviewed this information product for unauthorized disclosure of confidential information and have approved the disclosure avoidance practices applied to this release. This research was performed at a Federal Statistical Research Data Center under FSRDC Project Number 1284. (CBDRB-FY20-433, CBDRB-FY20-P1284-R8653, CBDRB-FY20-P1284-R8649, CBDRB-FY22-P1284-R9528 CBDRB-FY22-P1284-R9618, CBDRB-FY23-P1284-10670, and CBDRB-FY23-P1284-10742).
(TIF)

S1 Table Results for education components, at age 25. (PDF)

S2 Table Outcomes for general index, linear model (unweighted). Each grid cell represents a separate regression. Regressions include county-specific time trends, month-of-birth fixed effects, race, sex, and age. In contrast to Table 1, the “unweighted” results omit weights in the estimation and the “no county-level controls” results omit county of birth demographic controls. The Census Bureau’s Disclosure Review Board and Disclosure Avoidance Officers have reviewed this information product for unauthorized disclosure of confidential information and have approved the disclosure avoidance practices applied to this release. This research was performed at a Federal Statistical Research Data Center under FSRDC Project Number 1284. (CBDRB-FY20-433, CBDRB-FY20-P1284-R8653, CBDRB-FY20-P1284-R8649, CBDRB-FY22-P1284-R9528 CBDRB-FY22-P1284-R9618, CBDRB-FY23-P1284-10670, and CBDRB-FY23-P1284-10742).
(PDF)

S3 Table Outcomes conditional on working, linear model (unweighted). Each grid cell represents a separate regression. Regressions include county-specific time trends, month-of-birth fixed effects, race, sex, and age. In contrast to Table 1, the “unweighted” results omit weights in the estimation and the “no county-level controls” results omit county of birth demographic controls. The Census Bureau’s Disclosure Review Board and Disclosure Avoidance Officers have reviewed this information product for unauthorized disclosure of confidential information and have approved the disclosure avoidance practices applied to this release. This research was performed at a Federal Statistical Research Data Center under FSRDC Project Number 1284. (CBDRB-FY20-433, CBDRB-FY20-P1284-R8653, CBDRB-FY20-P1284-R8649, CBDRB-FY22-P1284-R9528 CBDRB-FY22-P1284-R9618, CBDRB-FY23-P1284-10670, and CBDRB-FY23-P1284-10742).
(PDF)

S4 Table Outcomes for general index, linear model (no county-level controls). Each grid cell represents a separate regression. Regressions include county-specific time trends, month-of-birth fixed effects, race, sex, and age. In contrast to Table 1, the “unweighted” results omit weights in the estimation and the “no county-level controls” results omit county of birth demographic controls. The Census Bureau’s Disclosure Review Board and Disclosure Avoidance Officers have reviewed this information product for unauthorized disclosure of confidential information and have approved the disclosure avoidance practices applied to this release. This research was performed at a Federal Statistical Research Data Center under FSRDC Project Number 1284. (CBDRB-FY20-433, CBDRB-FY20-P1284-R8653, CBDRB-FY20-P1284-R8649, CBDRB-FY22-P1284-R9528 CBDRB-FY22-P1284-R9618, CBDRB-FY23-P1284-10670, and CBDRB-FY23-P1284-10742).
(PDF)

S5 Table Outcomes conditional on working, linear model (no county-level controls). Each grid cell represents a separate regression. Regressions include county-specific time trends, month-of-birth fixed effects, race, sex, and age. In contrast to Table 1, the “unweighted” results omit weights in the estimation and the “no county-level controls” results omit county of birth demographic controls. The Census Bureau’s Disclosure Review Board and Disclosure Avoidance Officers have reviewed this information product for unauthorized disclosure of confidential information and have approved the disclosure avoidance practices applied to this release. This research was performed at a Federal Statistical Research Data Center under FSRDC Project Number 1284. (CBDRB-FY20-433, CBDRB-FY20-P1284-R8653, CBDRB-FY20-P1284-R8649, CBDRB-FY22-P1284-R9528 CBDRB-FY22-P1284-R9618, CBDRB-FY23-P1284-10670, and CBDRB-FY23-P1284-10742).
(PDF)

S6 Table Coefficients, quadratic model, for outcomes in general index. Each grid cell represents a separate regression. Regressions include county-specific time trends, county-year demographic averages, month-of-birth fixed effects, race, sex, and age. Weights account for Census sampling probabilities and the probability of being matched to unique birth county. F-test is for joint significance of lead variables. The Census Bureau’s Disclosure Review Board and Disclosure Avoidance Officers have reviewed this information product for unauthorized disclosure of confidential information and have approved the disclosure avoidance practices applied to this release. This research was performed at a Federal Statistical Research Data Center under FSRDC Project Number 1284. (CBDRB-FY20-433, CBDRB-FY20-P1284-R8653, CBDRB-FY20-P1284-R8649, CBDRB-FY22-P1284-R9528 CBDRB-FY22-P1284-R9618, CBDRB-FY23-P1284-10670, and CBDRB-FY23-P1284-10742).
(PDF)

S7 Table Coefficients, quadratic model, for outcomes conditional on working. Each grid cell represents a separate regression. Regressions include county-specific time trends, county-year demographic averages, month-of-birth fixed effects, race, sex, and age. Weights account for Census sampling probabilities and the probability of being matched to unique birth county. F-test is for joint significance of lead variables. The Census Bureau’s Disclosure Review Board and Disclosure Avoidance Officers have reviewed this information product for unauthorized disclosure of confidential information and have approved the disclosure avoidance practices applied to this release. This research was performed at a Federal Statistical Research Data Center under FSRDC Project Number 1284. (CBDRB-FY20-433, CBDRB-FY20-P1284-R8653, CBDRB-FY20-P1284-R8649, CBDRB-FY22-P1284-R9528 CBDRB-FY22-P1284-R9618, CBDRB-FY23-P1284-10670, and CBDRB-FY23-P1284-10742).
(PDF)

S8 Table Coefficients, cubic model, for outcomes in general index. Each grid cell represents a separate regression. Regressions include county-specific time trends, county-year demographic averages, month-of-birth fixed effects, race, sex, and age. Weights account for Census sampling probabilities and the probability of being matched to unique birth county. F-test is for joint significance of lead variables. The Census Bureau’s Disclosure Review Board and Disclosure Avoidance Officers have reviewed this information product for unauthorized disclosure of confidential information and have approved the disclosure avoidance practices applied to this release. This research was performed at a Federal Statistical Research Data Center under FSRDC Project Number 1284. (CBDRB-FY20-433, CBDRB-FY20-P1284-R8653, CBDRB-FY20-P1284-R8649, CBDRB-FY22-P1284-R9528 CBDRB-FY22-P1284-R9618, CBDRB-FY23-P1284-10670, and CBDRB-FY23-P1284-10742).
(PDF)

S9 Table Coefficients, cubic model, for outcomes conditional on work. Each grid cell represents a separate regression. Regressions include county-specific time trends, county-year demographic averages, month-of-birth fixed effects, race, sex, and age. Weights account for Census sampling probabilities and the probability of being matched to unique birth county. F-test is for joint significance of lead variables. The Census Bureau’s Disclosure Review Board and Disclosure Avoidance Officers have reviewed this information product for unauthorized disclosure of confidential information and have approved the disclosure avoidance practices applied to this release. This research was performed at a Federal Statistical Research Data Center under FSRDC Project Number 1284. (CBDRB-FY20-433, CBDRB-FY20-P1284-R8653, CBDRB-FY20-P1284-R8649, CBDRB-FY22-P1284-R9528 CBDRB-FY22-P1284-R9618, CBDRB-FY23-P1284-10670, and CBDRB-FY23-P1284-10742).
(PDF)

S1 Appendix (PDF)
