# Supplementary material for: Impact of in utero airborne lead exposure on long-run adult socio-economic outcomes: A population analysis using U.S. survey and administrative data
Source: PLoS One. 2023 Nov 22;18(11):e0293443. doi: 10.1371/journal.pone.0293443 (PMC10664929; doi:10.1371/journal.pone.0293443)
Supplement: S1 Table — (PDF) [file pone.0293443.s002.pdf]

S1 Table. Results for Education Components, at Age 25.

	Graduated from HS at 25	Graduated from Col. at 25
Lead	-0.004763	-0.009513
SE	(0.006195)	(0.01268)
Adjusted R^2	0.0446	0.1058
N (rounded)	184000	184000

The Census Bureau's Disclosure Review Board and Disclosure Avoidance Officers have reviewed this information product for unauthorized disclosure of confidential information and have approved the disclosure avoidance practices applied to this release. This research was performed at a Federal Statistical Research Data Center under FSRDC Project Number 1284. (CBDRB-FY20-433, CBDRB-FY20-P1284-R8653, CBDRB-FY20-P1284-R8649, CBDRB-FY22-P1284-R9528 CBDRB-FY22-P1284-R9618, CBDRB-FY23-P1284-10670, and CBDRB-FY23-P1284-10742.)
