# Supplementary material for: Impact of in utero airborne lead exposure on long-run adult socio-economic outcomes: A population analysis using U.S. survey and administrative data
Source: PLoS One. 2023 Nov 22;18(11):e0293443. doi: 10.1371/journal.pone.0293443 (PMC10664929; doi:10.1371/journal.pone.0293443)
Supplement: S2 Table — Each grid cell represents a separate regression. Regressions include county-specific time trends, month-of-birth fixed effects, race, sex, and age. In contrast to Table 1, the “unweighted” results omit weights in the estimation and the “no county-level controls” results omit county of birth demographic controls. The Census Bureau’s Disclosure Review Board and Disclosure Avoidance Officers have reviewed this information product for unauthorized disclosure of confidential information and have approved the disclosure avoidance practices applied to this release. This research was performed at a Federal Statistical Research Data Center under FSRDC Project Number 1284. (CBDRB-FY20-433, CBDRB-FY20-P1284-R8653, CBDRB-FY20-P1284-R8649, CBDRB-FY22-P1284-R9528 CBDRB-FY22-P1284-R9618, CBDRB-FY23-P1284-10670, and CBDRB-FY23-P1284-10742). (PDF) [file pone.0293443.s003.pdf]

S2 Table. Outcomes for General Index, Linear Model (Unweighted)

Dependent vars:	**Index 1 (Std Dev.)**	Inverse Hyperbolic Sine of Earnings	Negative of the No. of Disabilities	No Cognitive Disability	Worked Last Year	Not Unemploye d	No Public Assistance	Graduate from HS	Graduated from Col.
Lead	-0.02918***	-0.06023	-0.01268***	-0.003625*	-0.007193***	-0.003629*	-0.004842***	-0.002597	-0.005385
SE	(0.0104)	(0.03971)	(0.003726)	(0.002094)	(0.002516)	(0.001871)	(0.001793)	(0.002972)	(0.006938)
p-val	0.007	0.136	0.001	0.090	0.006	0.058	0.009	0.386	0.441
Adj p-val	na	0.128	0.011	0.099	0.023	0.079	0.023	0.199	0.199
R^2	0.0549	0.0454	0.0121	0.0109	0.0431	0.0160	0.0210	0.0423	0.0875
N (rounded)	280000	280000	280000	280000	280000	280000	280000	280000	280000

Each grid cell represents a separate regression. Regressions include county-specific time trends, month-of-birth fixed effects, race, sex, and age. In contrast to Table 1, the “unweighted” results omit weights in the estimation and the “no county-level controls” results omit county of birth demographic controls. The Census Bureau's Disclosure Review Board and Disclosure Avoidance Officers have reviewed this information product for unauthorized disclosure of confidential information and have approved the disclosure avoidance practices applied to this release. This research was performed at a Federal Statistical Research Data Center under FSRDC Project Number 1284. (CBDRB-FY20-433, CBDRB-FY20-P1284-R8653, CBDRB-FY20-P1284-R8649, CBDRB-FY22-P1284-R9528 CBDRB-FY22-P1284-R9618, CBDRB-FY23-P1284-10670, and CBDRB-FY23-P1284-10742.)
