# Supplementary material for: Impact of in utero airborne lead exposure on long-run adult socio-economic outcomes: A population analysis using U.S. survey and administrative data
Source: PLoS One. 2023 Nov 22;18(11):e0293443. doi: 10.1371/journal.pone.0293443 (PMC10664929; doi:10.1371/journal.pone.0293443)
Supplement: S3 Table — Each grid cell represents a separate regression. Regressions include county-specific time trends, month-of-birth fixed effects, race, sex, and age. In contrast to Table 1, the “unweighted” results omit weights in the estimation and the “no county-level controls” results omit county of birth demographic controls. The Census Bureau’s Disclosure Review Board and Disclosure Avoidance Officers have reviewed this information product for unauthorized disclosure of confidential information and have approved the disclosure avoidance practices applied to this release. This research was performed at a Federal Statistical Research Data Center under FSRDC Project Number 1284. (CBDRB-FY20-433, CBDRB-FY20-P1284-R8653, CBDRB-FY20-P1284-R8649, CBDRB-FY22-P1284-R9528 CBDRB-FY22-P1284-R9618, CBDRB-FY23-P1284-10670, and CBDRB-FY23-P1284-10742). (PDF) [file pone.0293443.s004.pdf]

S3 Table. Outcomes Conditional on working, Linear Model (Unweighted)

Dependent vars:	**Index 2 (Std Dev.)**	Ln Earnings, if >0	Ln Hourly wage, if >0	Worked full year (if working)	Worked full time (if working)	No. Weekly Hrs Usually Worked (if working)
Lead	-0.01055	-0.001097	0.01101	-0.008665**	-0.005008	-0.1868
SE	(0.0145)	(0.01142)	(0.008537)	(0.003620)	(0.005060)	(0.1257)
p-val	0.470	0.924	0.203	0.021	0.327	0.144
Adj p-val	n/a	0.587	0.372	0.115	0.487	0.372
R^2	0.0740	0.0678	0.0670	0.0209	0.0479	0.0808
N (rounded)	212000	212000	212000	212000	212000	212000

Each grid cell represents a separate regression. Regressions include county-specific time trends, month-of-birth fixed effects, race, sex, and age. In contrast to Table 1, the “unweighted” results omit weights in the estimation and the “no county-level controls” results omit county of birth demographic controls. The Census Bureau's Disclosure Review Board and Disclosure Avoidance Officers have reviewed this information product for unauthorized disclosure of confidential information and have approved the disclosure avoidance practices applied to this release. This research was performed at a Federal Statistical Research Data Center under FSRDC Project Number 1284. (CBDRB-FY20-433, CBDRB-FY20-P1284-R8653, CBDRB-FY20-P1284-R8649, CBDRB-FY22-P1284-R9528 CBDRB-FY22-P1284-R9618, CBDRB-FY23-P1284-10670, and CBDRB-FY23-P1284-10742.)
