# Supplementary material for: Impact of in utero airborne lead exposure on long-run adult socio-economic outcomes: A population analysis using U.S. survey and administrative data
Source: PLoS One. 2023 Nov 22;18(11):e0293443. doi: 10.1371/journal.pone.0293443 (PMC10664929; doi:10.1371/journal.pone.0293443)
Supplement: S6 Table — Each grid cell represents a separate regression. Regressions include county-specific time trends, county-year demographic averages, month-of-birth fixed effects, race, sex, and age. Weights account for Census sampling probabilities and the probability of being matched to unique birth county. F-test is for joint significance of lead variables. The Census Bureau’s Disclosure Review Board and Disclosure Avoidance Officers have reviewed this information product for unauthorized disclosure of confidential information and have approved the disclosure avoidance practices applied to this release. This research was performed at a Federal Statistical Research Data Center under FSRDC Project Number 1284. (CBDRB-FY20-433, CBDRB-FY20-P1284-R8653, CBDRB-FY20-P1284-R8649, CBDRB-FY22-P1284-R9528 CBDRB-FY22-P1284-R9618, CBDRB-FY23-P1284-10670, and CBDRB-FY23-P1284-10742). (PDF) [file pone.0293443.s007.pdf]

S6 Table. Coefficients, Quadratic Model, for Outcomes in General Index

Dependent vars:	**Index 1 (Std Dev.)**	Inverse Hyperbolic Sine of Earnings	Negative of the No. of Disabilities	No Cognitive Disability	Worked Last Year	Not Unemployed	No Public Assistance	Graduate from HS	Graduated from Col.
lead	-0.03814**	-0.1657***	-0.01571*	-0.006528	-0.01229*	-0.005779	-0.005814**	-0.001205	0.006605
SE	(0.0189)	(0.06100)	(0.009018)	(0.003981)	(0.006426)	(0.004400)	(0.002832)	(0.004722)	(0.008913)
Lead squared	0.0016	0.02212***	0.0007039	0.0002230	0.0008737	0.0003756	0.0002564	-0.0004465	-0.001631*
SE	(0.0024)	(0.007379)	(0.001483)	(0.0003887)	(0.0008715)	(0.0007771)	(0.0003086)	(0.0008474)	(0.0009235)
F-Test	3.27	4.69	2.30	2.84	4.19	1.44	2.19	0.34	7.94
R^2	0.0505	0.0420	0.0132	0.0117	0.0403	0.0214	0.0252	0.0414	0.0902
N (rounded)	280000	280000	280000	280000	280000	280000	280000	280000	280000

Each grid cell represents a separate regression. Regressions include county-specific time trends, county-year demographic averages, month-of-birth fixed effects, race, sex, and age. Weights account for Census sampling probabilities and the probability of being matched to unique birth county. F-test is for joint significance of lead variables. The Census Bureau's Disclosure Review Board and Disclosure Avoidance Officers have reviewed this information product for unauthorized disclosure of confidential information and have approved the disclosure avoidance practices applied to this release. This research was performed at a Federal Statistical Research Data Center under FSRDC Project Number 1284. (CBDRB-FY20-433, CBDRB-FY20-P1284-R8653, CBDRB-FY20-P1284-R8649, CBDRB-FY22-P1284-R9528 CBDRB-FY22-P1284-R9618, CBDRB-FY23-P1284-10670, and CBDRB-FY23-P1284-10742.)
