# Supplementary material for: Impact of in utero airborne lead exposure on long-run adult socio-economic outcomes: A population analysis using U.S. survey and administrative data
Source: PLoS One. 2023 Nov 22;18(11):e0293443. doi: 10.1371/journal.pone.0293443 (PMC10664929; doi:10.1371/journal.pone.0293443)
Supplement: S1 Appendix — (PDF) [file pone.0293443.s011.pdf]

S10. Appendix[1]

Discussion of county-level lead measures

As discussed in the text, we assign county-level air lead measures lagged over 267 days to all people born in a given county on a given day. The county-level lead measures are averaged up from local measures, weighting by population. When weighted by population in this way, county-level pollution measures are arguably more relevant than more local measures, if the mother travels away from home to work, to conduct errands, or for other reasons, and if such travel gravitates to population centers. Consider the following model. Suppose each mother i spends α_i proportion of her time at home and $(1 - \alpha_i)$ away from home, at a mix of locations within a county that are also weighted by population. That is, mothers systematically have travel patterns that gravitate to population centers, so somebody living in a rural area is more likely to go to a city for errands, recreation and so forth, than somebody in the city is likely to go the countryside. Then, if L_i is the lead exposure at i's home and L is the county-wide population-weighted average, a mother's true exposure is $\alpha_i L_i + (1 - \alpha_i)L$. Finally, assume this parameter α_i is independent of deviations of i's home from county-average pollution, that is, $E[\alpha_i(L_i - L)] = 0$. If we had taken the county-wide average of these true measures instead of the average of values only at home, we would have obtained $E[\alpha_i L_i + (1 - \alpha_i)L] = L + E[\alpha_i(L_i - L)] = L$. But this is precisely what we do measure! Thus, our measure is robust to plausible forms of travel.

Of course, even within the logic of this model, each individual mother is actually exposed to a pollution level of $\alpha_i L_i + (1 - \alpha_i)L$, not the value L which we assign. But this remaining measurement error is Berkson, not classical. Whereas classical measurement error represents a situation where the observed variable is equal to the true underlying variable plus noise, Berkson measurement error represents the opposite situation, where the true variable is equal to the observed variable plus unobserved dispersion. It occurs, for example, from assigning group averages rather than individual data. As with classical measurement error in the dependent variable, Berkson measurement error in an independent variable increases standard errors but does not cause bias. To see why any errors in our lead measure are likely of the Berkson type, note that as long as we correctly identify the county of birth, and as long as homes of mothers are spatially distributed within a county in the same way as the overall population, then all births are assigned the "correct average" pollution value, where the correct average is taken over homes of mothers.

Calculation of economic benefits

To compute cumulative 1992-2020 impacts on earnings, we combined several data sources. First, in each year (1992-2020), we obtained total counts of individuals at each age, if born after 1975 and at least 16. For 1992-1999, we interpolated these numbers from the 1990 and 2000 US Census. For 2000, we used the latter. For 2001-3, we interpolated from the 2000 Census and the

[1] The Census Bureau's Disclosure Review Board and Disclosure Avoidance Officers have reviewed this information product for unauthorized disclosure of confidential information and have approved the disclosure avoidance practices applied to this release. This research was performed at a Federal Statistical Research Data Center under FSRDC Project Number 1284. (CBDRB-FY20-433, CBDRB-FY20-P1284-R8653, CBDRB-FY20-P1284-R8649, CBDRB-FY22-P1284-R9528 CBDRB-FY22-P1284-R9618, CBDRB-FY23-P1284-10670, and CBDRB-FY23-P1284-10742.)

2004 ACS. For 2005-2020, we used each year's annual ACS. Second, we obtained total wages and salaries for the United States in each year from the US National Income and Product Accounts (NIPA), using the Bureau of Economic Analysis's (Table WASCUR). Third, we obtained average earnings by age for each year from the US Census. For 1992-2003, these were obtained from the Bureau of Labor Statistics's Consumer Expenditure Survey (Series CXU900000LB04). These data are only available in 10-year age bins, which we split uniformly across all ages in the bin. For 2004, these data were obtained from the ACS, and were available for each age. These data were combined as explained in the text.

To convert our estimates of effects *in utero* lead exposure on current-year earnings to effects on *lifetime* earnings (vs. 1992-2020 earnings), we consider three life-cycle models. The first comes from an analysis of data from the 2004 Survey of Income and Program Participation (SIPP) matched to earnings records from the SSA [74]. It uses a 20-year panel of earnings for different age groups to construct a pseudo panel of lifetime earnings, constructing a present value (PV) at age 20. The study reports results by education level. We combine their results with population shares by education level to construct an overall average, then discount it to birth from age 20 using a discount rate of 3%. This results in a PV of $693,200. The second is from an analysis of the 2007-2009 ACS [75]. The study looks at earnings by age at a point in time and assumes that profile continues to hold for a cohort across time. It estimates the median PV of lifetime earnings for those 20-70 in 2007-9 to be $601,500 (when we use a discount rate of 3% rather than the 2.5% used in their study). The third estimate is our own analysis of the 2020 Current Population Survey (CPS) supplement, using the same methodology except using the mean rather than median, and our central discount rate of 3%. Our estimate is $534,500. Averaging the three gives a representative estimate of $609,700. Using our estimated effect on earnings of 7.01% per μg/m, combined with the permanent decrease in lead 0f 0.5 $\mu g/m^3$, then gives a PV, at birth, of $21,400 per capita.
